# Cardiometabolic Risk in Hyperlipidemic Men and Women

**DOI:** 10.1155/2016/2647865

**Published:** 2016-11-08

**Authors:** Michael Leutner, Christian Göbl, Alice Wielandner, Eleonora Howorka, Marlies Prünner, Latife Bozkurt, Jürgen Harreiter, Helmut Prosch, Oliver Schlager, Silvia Charwat-Resl, Alexandra Kautzky-Willer

**Affiliations:** ^1^Department of Internal Medicine III, Division of Endocrinology and Metabolism, Unit of Gender Medicine, Medical University of Vienna, Waehringer Guertel 18-20, 1090 Vienna, Austria; ^2^Department of Gynecology and Obstetrics, Division of Feto-Maternal Medicine, Medical University of Vienna, Waehringer Guertel 18-20, 1090 Vienna, Austria; ^3^Department of Biomedical Imaging and Image Guided Therapy, Medical University of Vienna, Waehringer Guertel 18-20, 1090 Vienna, Austria; ^4^Department of Internal Medicine III, Clinical Division of Endocrinology and Metabolism, Medical University of Vienna, Waehringer Giertel 18-20, 1090 Vienna, Austria; ^5^Department of Internal Medicine II, Division of Angiology, Medical University of Vienna, Waehringer Guertel 18-20, 1090 Vienna, Austria

## Abstract

*Objective*. The aim of this study was to evaluate sex specific differences of metabolic and clinical characteristics of treated hyperlipidemic men and women (HL-men and HL-women).* Methods*. In this study vascular and metabolic characteristics of 35 HL-women and 64 HL-men were assessed. In addition a sex specific analysis of metabolic and nutritional habits of HL-patients with prediabetes (HL-IGR) was done.* Results*. HL-women were older and had favourable concentrations of high density lipoprotein cholesterol (HDL-cholesterol), triglycerides (TG), and triglyceride/HDL-cholesterol ratio (TG/HDL-ratio) but were also shown to have higher concentrations of lipoprotein-a compared to HL-men. HL-men were characterized as having higher levels of liver-specific parameters and body weight as well as being more physically active compared to HL-women. Brain natriuretic peptide (pro-BNP) was higher in HL-women than HL-men, while no differences in metabolic syndrome and glycemic parameters were shown. HL-IGR-women were also older and still had a better profile of sex specific lipid parameters, as well as a lower body weight compared to HL-IGR-men. No differences were seen in vascular parameters such as the intima media thickness (IMT).* Conclusion*. HL-women were older and had overall more favourable concentrations of lipid parameters and liver enzymes but did not differ regarding vascular morphology and insulin sensitivity compared to HL-men of comparable body mass index (BMI).

## 1. Background

In general men have a higher risk of cardiovascular mortality compared to women [1]. In addition to the better outcome in cardiovascular disease (CVD) in women, the time of occurrence of CVD is also later [2]. These discrepancies occur due to the significant influence that sex plays on the development of CVD [1].

One of the major influences on CVD is the lipid profile [3, 4] and it is well known that there are sex specific differences in the progression of CVD affected by hyperlipidemia [5, 6]. In addition studies have shown that menopause and female sex hormones have a big influence on lipid parameters [7]. Studies show that there are also sex specific differences in the reach of target values of lipid parameters. Wenger showed that hyperlipidemic men reach more often the target values of blood parameters compared to women [8].

These discrepancies occur because there are multifactorial sex specific factors which influence the reach of target [9, 10]. Nevertheless, dyslipidemic treatment reduces the occurrence of CVD in both sexes [11, 12].

In addition to dyslipidemia, prediabetes is also a risk factor for the development of CVD [13]. Studies show that women with high blood glucose levels have a higher risk of cardiovascular mortality compared to men [14–17].

Little is known about the combination of hyperlipidemia with hyperglycemia.

It was shown that there is a cumulative effect of hyperlipidemia and prediabetes on atherosclerosis and the development of coronary heart disease [18, 19].

Therefore, further investigations are necessary. The objective of this analysis is to compare the metabolic and clinic characteristics of hyperlipidemic men and women with and without prediabetes.

## 2. Methods

The detailed study procedures were previously described [18]. The primary outcome of this study was to compare sex specific differences in treated hyperlipidemic patients. We also studied sex differences in the high risk subgroup with prediabetes in this analysis.

In brief, this study included 35 women and 64 men with hyperlipidemia. The subanalysis of hyperlipidemic patients with prediabetes consisted of 19 women and 29 men. All patients were diagnosed and treated according to the ESC/EAS guidelines [20].

Prediabetes was diagnosed with glycated hemoglobin A1c (HbA1c) levels of ≥5.7% and <6.5% or/and fasting glucose levels of ≥100 mg/dL and <126 mg/dL according to the guidelines of the American Diabetes Association [21].

Inclusion criteria were an age between 35 and 75 years and a consistent dyslipidemic treatment (including diet), for at least the last three months before participating in the study. Patients with diabetes mellitus type-1 or type-2 were excluded from this study [18]. The study participants were divided into two groups according to sex.

Patients had a positive history of CVD if they had been diagnosed with one or more of the following: stroke, coronary heart disease, peripheral artery disease, myocardial infarction, or angina pectoris. Data from a 7-day food intake diary was used for the analysis of the mean daily energy intake (analysed with the program nut.s, nutritional software v1.32.37-2015.12.15, URL: http://www.nutritional-software.at/) in the specific subpopulation of HL-IGR-men and HL-IGR-women. The medical history of the patients was assessed by a questionnaire and anthropometric data such as height, body weight, or waist circumference, as well as the blood pressure, were measured according to standardized procedures [18].

Physical activity was measured using an Omron Walking Style II pedometer. Therefore, study participants were instructed to take the pedometer for 7 days. After the 7 days the mean steps of the pedometer were calculated [18].

### 2.1. IMT Measurements

IMT measurements were performed by ultrasound and conducted by two experienced coinvestigators according to standardized procedures as previously described [18]. Measurements were obtained by using a 9 MHz linear transducer probe of an Acuson ultrasound machine (Acuson XP 128, Siemens Medical Solutions, USA). IMT was measured by using frozen B-mode images of the far wall of the distal common carotid artery, approximately 1.5–2 cm proximal to the carotid bifurcation. In terms of reaching the values of “good clinical practice” the analysation process was blinded and performed under the supervision of an expert coinvestigator and by an experienced coinvestigator [18].

### 2.2. Laboratory Measurements

As previously described [18], laboratory parameters were taken in fasting conditions (≥10 hours) and measurements, including serum lipids, fasting plasma glucose, fasting insulin, C-peptide, HbA1c, parameters of liver function, pro-BNP, and high sensitive CRP (hsCRP), were analysed according to international standard laboratory methods at the Department of Medical and Chemical Laboratory Diagnostics (http://www.kimcl.at/).

In accordance with the general guidelines the Friedewald formula was used for the assessment of low density lipoprotein cholesterol (LDL-cholesterol) [22].

### 2.3. Calculations

We measured insulin sensitivity by using the QUICKI-test [23].

### 2.4. Statistical Analysis

Means ± standard deviations were stated for normal continuous variables and percentages for categorical variables. Median and interquartile range were stated if variables were not normally distributed (verification with the Shapiro-Wilk-test).

Students *t*-test and Fisher's exact test were applied for sex specific group comparisons (men versus women). If normality assumption was not given, Wilcoxon rank sum test was used for group based comparisons. Logarithmic transformation was used for parameters which were strongly skewed: ln(TG), ln(TG/HDL-ratio), ln(lipoprotein-a), ln(insulin+1), ln(pro-BNP), ln(hsCRP+1), ln(glutamate-oxaloacetate transaminase, GOT), ln(glutamate-pyruvate transaminase, GPT), and ln(gamma-glutamyl transferase, GGT). Pearson's product moment correlation was used for the analysis of correlations between continuous variables.

In the case of multivariable adjustment linear two-way ANOVA models (modeling interactions between sex and prediabetes on lipid parameters) were used.

Power analysis was performed to determine the sample size needed to achieve 80% test power for the *t*-test analyses. This analysis was performed using G^*∗*^Power software, version 3.1.9.2 (software freely available from the University of Düsseldorf). For fasting glucose, a mean difference of 10 mg/dL and a standard deviation of 14 mg/dL were assumed as clinically relevant. Therefore the minimal sample size was calculated as 32 patients per group. For LDL-cholesterol, for a mean difference of 30 mg/dL and assumed standard deviation of 40 mg/dL, the minimal sample size was calculated as 29 patients per group.

The statistical analysis was done with R (V3.1.1) [24]. Statistical significance was assumed with a two-sided *p* value of ≤0.05.

## 3. Results

In this study 35 HL-women and 64 HL-men were included. A descriptive comparison of study participants, divided by sex, is shown in Table 1. This table shows that HL-women were older and had a lower body weight and waist circumference but did not differ regarding BMI and the systolic and diastolic blood pressure compared to HL-men. HL-women were shown to have better concentrations of lipid parameters such as HDL-cholesterol, TG, and TG/HDL-ratio but had higher concentrations of lipoprotein-a compared to HL-men (Table 1). No sex specific difference was shown in the occurrence of LDL-cholesterol levels < 100 mg/dL. Univariable comparisons showed that there was no significant difference in carotid IMT measurements between HL-men and HL-women. In addition, no sex specific differences in the glucose metabolism (glucose, insulin, C-peptide, HbA1c, and QUICKI-test) were found. HL-women were also shown to have higher levels of traditional cardiovascular risk marker pro-BNP, but not hs-CRP (Table 1).

HL-men in this study were characterized as being more physically active (mean steps in 7 days) and as having significantly higher liver-specific parameters (GGT, GPT). No sex specific differences were observed in the occurrence of a history of CVD and of the metabolic syndrome (Table 1).

## 4. Sex Specific Subanalysis of HL-Patients with Prediabetes Including Nutritional Data

In addition to the analysis above, a subanalysis of 19 women and 29 men with hyperlipidemia additionally affected by a prediabetes was done. A descriptive comparison of study participants, divided by sex, is shown in Table 2.

This table shows that HL-IGR-women were older and had a lower body weight and waist circumference but did not differ in other anthropometric parameters such as BMI and the systolic and diastolic blood pressure compared to HL-IGR-men. There was no significant difference in modifiable lipid parameters (total cholesterol, LDL-cholesterol, and TG) between sexes. HL-IGR-women were shown to have significantly higher levels of HDL-cholesterol and lipoprotein-a, as well as favourable concentrations of TG/HDL-ratio compared to HL-IGR-men. No difference was found in carotid IMT measurements between HL-IGR-men and HL-IGR-women. In addition, no differences in glucose metabolism (glucose, insulin, C-peptide, HbA1c, and QUICKI-test) and in cardiovascular risk markers (pro-BNP, hsCRP) between sexes were found in this specific subanalysis (Table 2).

HL-IGR-men were shown to have higher liver-specific parameters (GGT, Table 2).  Table 3 shows that glycogen, vitamin-B12, and protein intake, measured with a dietary protocol, were significantly higher in HL-IGR-men.

As shown in Table 4 sex specific analyses revealed that waist circumference is related to concentrations of TG in HL-IGR-women, but not in HL-IGR-men. No association with waist circumference was observed for any other lipid parameters (LDL-cholesterol, HDL-cholesterol, total cholesterol, and TG in men) or parameters of glucose metabolism (fasting plasma glucose, HbA1c) in both sexes. In addition no correlation of waist circumference with IMT in both sexes was found. Table 4 also shows that there was no association for body weight and BMI with lipid parameters and parameters of glucose metabolism in HL-IGR-men and HL-IGR-women. In general no interactions between sex and prediabetes with lipid parameters (LDL-cholesterol, HDL-cholesterol, TG, and total cholesterol) were found.

## 5. Discussion

HL-women are characterized as being older and having a lower body weight and waist circumference, more favourable concentrations of HDL-cholesterol, TG, and TG/HDL-ratio, and better concentrations of liver enzymes, but they do not differ regarding insulin sensitivity and the IMT compared to HL-men. The metabolic characteristics of HL-IGR-men and HL-IGR-women are in general comparable with the metabolic characteristics of the whole study population.

The fact that HL-women have favourable concentrations of certain lipid parameters (HDL-cholesterol, TG/HDL-ratio) is a well described sex specific difference in various conditions. In general men have a higher risk of CVD compared to women [1]. The higher levels of HDL-cholesterol in women are one of the major protective factors against developing CVD, which leads to a later occurrence of cardiovascular disease in women [2]. Female sex hormones relate to higher HDL-cholesterol levels in women and exert overall protective effects against CVD [25, 26]. In our study population there were no significant sex specific differences in modifiable lipid parameters, such as LDL-cholesterol or total cholesterol. Only the concentrations of TG were shown to be significantly lower in female participants. This is an interesting point because studies showed that women were less likely to reach treatment goals of lipid parameters compared to men [27–29]. So the similarity of lipid parameters, such as LDL-cholesterol or total cholesterol between the sexes in our study, could point out that the previously described sex specific differences in reach of target values of lipid parameters faded. To support this hypothesis our results showed that there is no sex specific difference in the presence of LDL-cholesterol levels < 100 mg/dL in this study. The fact that HL-women have higher concentrations of cardiovascular risk marker pro-BNP could point to a higher risk for CVD. Wang et al. showed that women have in general higher concentrations of natriuretic peptide (NP) [30], which positively correlate with estrogen concentrations [31, 32]. Lam et al. also showed that women in menopause have still higher concentrations of NP compared to men. Additionally they showed that testosterone has a decreasing effect on the concentrations of NP in men [33]. Nevertheless Kannel showed that women in the postmenopausal status have a significant increase in the development of CVD [34]. So larger and longitudinal studies are required in order to find conclusive results, whether the higher concentration of pro-BNP is due to sex hormones in this specific population.

In general the metabolic characterization of the high risk population of HL-IGR-women and men is comparable with the results of the whole study population above.

Although there were not any significant sex specific differences in traditional cardiovascular risk markers in the specific population of HL-IGR-patients, it can be speculated that the cohort of HL-IGR-women is at higher risk for mortality compared to HL-IGR-men because in addition to hyperlipidemia, hyperglycemia is, especially in women, a higher risk factor for cardiovascular mortality compared to men [15–17].

Therefore, it has to be reported that atherogenic dyslipidemia, including reduced levels of HDL-cholesterol and increased levels of small dense LDL particles and triglycerides, is a feature of prediabetes. This interrelationship between impaired glucose regulation and lipid abnormalities leads in further consequence to an increased risk of developing CVD, indicating that HL-IGR-patients could be a high risk population [35].

Despite the lack of significance regarding the occurrence of CVD, men showed a higher occurrence rate (29.6%) compared to women (5.6%) in this study. This number seems to be plausible because men have in general a higher risk of developing CVD [1] with an earlier onset [2]. Nevertheless the specific subpopulation of treated HL-IGR-women around menopause could be a high risk population which should be monitored and treated to target more thoroughly. Interestingly in our study it was shown that there is a positive correlation of triglycerides with waist circumference in HL-IGR-women, but not in men. So an adverse body composition could be a higher risk factor for metabolic diseases in HL-IGR-women. Therefore, larger studies are required to investigate these sex specific differences.

One major limitation of this study is the low number of participants and that prediabetes was diagnosed using fasting blood parameters and not the oral glucose tolerance test. In addition the difference in the mean ages is a further limitation.

We are aware that with our study design smaller effects could have been missed. This study has an exploratory character and should show the sex specific cardiometabolic characteristics in the specific population of treated hyperlipidemic men and women. Nevertheless a greater number of study participants would be needed in order to investigate the real risk of this specific population.

In conclusion HL-women were older and had overall more favourable concentrations of lipid parameters and liver enzymes but did not differ regarding vascular morphology and insulin sensitivity compared to men of comparable BMI. Nevertheless the age of menopause could be a major risk factor for CVD. So HL-women, especially after menopause, should be treated and observed more carefully, not only regarding hyperlipidemia, but also regarding hyperglycemia. Larger studies should be done in order to evaluate the real risk of this specific subpopulation.

## Figures and Tables

**Table 1:** Characteristics of the study sample, divided by sex.

	*n* (female/male)	Female	Male	*p* value
Age [years]^*∗*^	35/64	58.0 ± 10.8	50.4 ± 9.3	0.001
BMI [kg/m^2^]^*∗*^	34/61	27.7 ± 5.5	27.7 ± 3.5	0.998
Body weight	34/61	72.99 ± 13.72	88.47 ± 12.62	<0.001
Waist [cm]^*∗*^	31/58	91.8 ± 11.6	98.7 ± 12.2	0.011
RRS [mmHg]^*∗*^	24/45	125.9 ± 14.4	132.2 ± 14.8	0.089
RRD [mmHg]^*∗*^	24/45	78.5 ± 8.7	81.8 ± 10.4	0.162
TC [mg/dL]^*∗*^	35/64	243.9 ± 72.3	227.6 ± 74.1	0.290
LDL-C [mg/dL]^*∗*^	34/60	143.3 ± 59.2	127.1 ± 45.6	0.174
LDL-C < 100 mg/dL	34/60	9 (26.5%)	17 (28.3%)	0.911
HDL-C [mg/dL]^*∗*^	33/60	65.2 ± 19.6	45.0 ± 15.1	<0.001
NHDL-C [mg/dL]^*∗*^	33/60	173.1 ± 61.5	172.2 ± 59.3	0.947
ln(TG) [mg/dL]^*∗*^	35/64	5.1 ± 0.64	5.53 ± 0.82	0.008
ln(TG/HDL)^*∗*^	33/60	0.89 ± 0.7	1.65 ± 0.87	<0.001
ln(Lip.a) [units]^*∗*^	30/57	3.5 ± 1.05	2.94 ± 1.22	0.031
IMT-left [mm]^*∗*^	31/58	0.65 ± 0.18	0.63 ± 0.14	0.584
IMT-right [mm]^*∗*^	30/56	0.64 ± 0.15	0.63 ± 0.12	0.824
IMT av [mm]^*∗*^	30/56	0.64 ± 0.14	0.63 ± 0.12	0.711
Glucose [mg/dL]^*∗*^	34/61	93.4 ± 10.8	94.2 ± 10.7	0.720
ln(insulin+1) [*μ*U/mL]^*∗*^	32/60	2.05 ± 0.69	2.28 ± 0.67	0.131
C-peptide [ng/mL]^*∗*^	28/50	2.91 ± 2.07	2.65 ± 1.11	0.546
HbA1c [%]^*∗*^	35/62	5.61 ± 0.37	5.46 ± 0.38	0.054
QUICKI-test^*∗*^	29/49	0.37 ± 0.05	0.35 ± 0.04	0.266
ln(proBNP) [units]^*∗*^	30/55	4.57 ± 0.79	3.77 ± 1.20	<0.001
ln(hsCRP+1) [units]^*∗*^	30/62	0.30 ± 0.28	0.21 ± 0.21	0.161
ln(GOT) [mg/dL]^*∗*^	34/64	3.29 ± 0.37	3.38 ± 0.35	0.263
ln(GPT) [mg/dL]^*∗*^	34/64	3.22 ± 0.47	3.46 ± 0.47	0.021
ln(GGT) [mg/dL]^*∗*^	34/64	3.28 ± 0.76	3.79 ± 0.82	0.003
Prediabetes^*∗∗*^	34/62	19 (55.9)	29 (46.8)	0.522
Ezetimibe^*∗∗*^	33/55	3 (9.1)	6 (10.9)	1.000
Nicotinic acid^*∗∗*^	33/54	1 (3.0)	4 (7.4)	0.646
Fibrates^*∗∗*^	33/54	5 (15.2)	22 (40.7)	0.017
Statins^*∗∗*^	33/54	16 (48.5)	38 (70.4)	0.068
CVD^*∗∗*^	33/60	2 (6.1)	10 (16.7)	0.202
Mean steps 7 days^*∗*^	31/47	7230.2 ± 2106.1	8500.1 ± 3395.9	0.045
Metabolic syndrome^*∗∗*^	21/44	7 (33.3)	16 (36.4)	1.000
Mean treatment duration of the last taken dyslipidemic medication (days)^*∗*^	33/62	2068.03 ± 2635.61	1879.77 ± 1997.47	0.721

^*∗*^ *t*-test, ^*∗∗*^Chi-square test.

Data are presented as number of observations (*n*) and means ± standard deviation. BMI (body mass index), waist (waist circumference), RRS (systolic blood pressure), RRD (diastolic blood pressure), TC (total cholesterol), LDL-C (low density lipoprotein cholesterol), HDL-C (high density lipoprotein cholesterol), NHDL-C (non-high density lipoprotein cholesterol), TG (triglycerides), TG/HDL (triglyceride/HDL-cholesterol ratio), Lip.a (lipoprotein (a)), IMT (carotid intima media thickness), HbA1c (glycated hemoglobin A1c), QUICKI (quantitative insulin sensitivity check index), proBNP (pro B-type natriuretic peptide), hsCRP (high sensitive C-reactive protein), GOT (glutamate-oxaloacetate transaminase), GPT (glutamate-pyruvate transaminase), GGT (gamma-glutamyl transferase), and CVD (cardiovascular disease).

**Table 2:** Characteristics of the study sample with prediabetes, divided by sex.

	*n* (female/male)	Female	Male	*p* value
Age [years]^*∗*^	19/29	62.4 ± 9.6	54.4 ± 9.8	0.007
Body weight	19/28	73.50 ± 13.62	90.49 ± 13.55	<0.001
BMI [kg/m^2^]^*∗*^	19/28	27.8 ± 4.8	28.9 ± 3.7	0.360
Waist [cm]^*∗*^	18/27	94.9 ± 9.2	104.4 ± 8.9	0.001
RRS [mmHg]^*∗*^	14/20	127.5 ± 17.6	136.4 ± 18.2	0.164
RRD [mmHg]^*∗*^	14/20	77.2 ± 10.7	82.1 ± 11.5	0.223
TC [mg/dL]^*∗*^	19/29	238.6 ± 68.3	231.6 ± 81.5	0.371
LDL-C [mg/dL]^*∗*^	18/27	136.2 ± 50.2	131.5 ± 45.4	0.746
HDL-C [mg/dL]^*∗*^	17/27	62.7 ± 16.1	43.7 ± 14.6	<0.001
NHDL-C [mg/dL]^*∗*^	17/27	164.3 ± 47.4	171.5 ± 50.5	0.640
ln(TG) [mg/dL]^*∗*^	19/29	5.2 ± 0.69	5.55 ± 0.76	0.165
ln(TG/HDL)^*∗*^	17/27	0.97 ± 0.66	1.70 ± 0.81	0.003
ln(Lip.a) [units]^*∗*^	17/26	4.00 ± 1.04	2.96 ± 1.40	0.013
IMT-left [mm]^*∗*^	17/26	0.68 ± 0.18	0.69 ± 0.17	0.576
IMT-right [mm]^*∗*^	17/26	0.69 ± 0.16	0.66 ± 0.14	0.463
IMT av [mm]^*∗*^	17/26	0.69 ± 0.15	0.68 ± 0.14	0.834
Glucose [mg/dL]^*∗*^	19/27	99.8 ± 9.2	100.3 ± 12.0	0.882
ln(insulin+1) [*μ*U/mL]^*∗*^	17/27	2.15 ± 0.77	2.41 ± 0.75	0.273
C-peptide [ng/mL]^*∗*^	16/23	3.44 ± 2.55	3.03 ± 1.16	0.656
HbA1c [%]^*∗*^	19/28	5.83 ± 0.32	5.73 ± 0.33	0.322
QUICKI-test^*∗*^	16/23	0.36 ± 0.05	0.35 ± 0.05	0.478
ln(proBNP) [units]^*∗*^	16/23	4.18 ± 0.61	4.23 ± 1.51	0.905
ln(hsCRP+1) [units]^*∗*^	17/28	0.30 ± 0.29	0.20 ± 0.17	0.440
ln(GOT) [mg/dL]^*∗*^	18/29	3.34 ± 0.35	3.41 ± 0.37	0.499
ln(GPT) [mg/dL]^*∗*^	18/29	3.32 ± 0.45	3.55 ± 0.46	0.098
ln(GGT) [mg/dL]^*∗*^	18/29	3.52 ± 0.78	3.96 ± 0.70	0.048
Ezetimibe^*∗∗*^	19/23	2 (10.5)	3 (13.0)	1.000
Nicotinic acid^*∗∗*^	19/23	0 (0.0)	2 (8.7)	0.556
Fibrates^*∗∗*^	19/23	2 (10.5)	9 (39.1)	0.081
Statins^*∗∗*^	19/23	11 (57.9)	19 (82.6)	0.155
CVD^*∗∗*^	18/27	1 (5.6)	8 (29.6)	0.110
Mean steps 7 days^*∗*^	17/21	7009.8 ± 2520.4	8381.4 ± 2660.0	0.114
Metabolic syndrome^*∗∗*^	12/20	5 (41.7)	12 (60.0)	0.522
Mean treatment duration of the last taken dyslipidemic medication (days)^*∗*^	18/28	2272.3 ± 2649.9	2132.8 ± 2362.1	0.938

^*∗*^ *t*-test, ^*∗∗*^Chi-square test.

Data are presented as number of observations (*n*) and means ± standard deviation. BMI (body mass index), waist (waist circumference), RRS (systolic blood pressure), RRD (diastolic blood pressure), TC (total cholesterol), LDL-C (low density lipoprotein cholesterol), HDL-C (high density lipoprotein cholesterol), NHDL-C (non-high density lipoprotein cholesterol), TG (triglycerides), TG/HDL (triglyceride/HDL-cholesterol ratio), Lip.a (lipoprotein (a)), IMT (carotid intima media thickness), HbA1c (glycated hemoglobin A1c), QUICKI (quantitative insulin sensitivity check index), proBNP (pro B-type natriuretic peptide), hsCRP (high sensitive C-reactive protein), GOT (glutamate-oxaloacetate transaminase), GPT (glutamate-pyruvate transaminase), GGT (gamma-glutamyl transferase), and CVD (cardiovascular disease).

**Table 3:** Nutritional characteristics of the study sample with prediabetes, divided by sex.

	*n* (female/male)	Female	Male	*p* value
Total energy intake (kcal)^*∗*^	14/20	1386.6 ± 388.9	1790.0 ± 693.6	0.104
Fat (mg)^*∗*^	14/20	53456.7 ± 20456.9	61750.4 ± 30408.8	0.381
Cholesterol (mg)^*∗*^	14/20	207 ± 99.4	297.4 ± 153.9	0.063
Omega-3 fatty acids (mg)^*∗*^	14/20	1700.8 ± 978.5	1767.6 ± 1032.6	0.796
Omega-6 fatty acids (mg)^*∗*^	14/20	7807.6 ± 3722.0	8100.2 ± 3762.5	0.824
Polyunsaturated fatty acids (mg)^*∗*^	14/20	9511.2 ± 4487.1	10284.0 ± 4786.2	0.638
Saturated fatty acids (mg)^*∗*^	14/20	22987.7 ± 10056.8	27342.2 ± 13537.4	0.315
Monounsaturated fatty acids (mg)^*∗*^	14/20	17529.6 ± 6988.6	29249.2 ± 30968.1	0.097
Carbohydrates (mg)^*∗*^	14/20	157769.7 ± 40805.0	177359.1 ± 79750.7	0.356
Disaccharides (mg)^*∗*^	14/20	38443.8 ± 18889.7	35274.7 ± 25976.0	0.457
Lactose (mg)^*∗*^	14/20	7232.8 ± 8305.5	6326.8 ± 7283.3	0.738
Sucrose (mg)^*∗*^	14/20	30101.6 ± 12942.5	28658.6 ± 23784.8	0.416
Monosaccharides (mg)^*∗*^	14/20	26831.4 ± 10530.5	27196.4 ± 17224.5	0.944
Fructose (mg)^*∗*^	14/20	15225.3 ± 6670.6	14812.5 ± 9728.1	0.892
Glucose (mg)^*∗*^	14/20	11032.1 ± 3862.8	11869.7 ± 7680.7	0.679
Glycogen (mg)^*∗*^	14/20	33.6 ± 56.1	239.0 ± 255.9	0.001
Vitamin B12 (*μ*g)^*∗*^	14/19	3.0 ± 1.6	4.6 ± 1.9	0.011
Dietary fiber (mg)^*∗*^	14/20	18009.2 ± 5366.7	20243.3 ± 10600.1	0.904
Protein (mg)^*∗*^	14/19	55761.9 ± 20497.3	77520.9 ± 27476.4	0.018

^*∗*^ *t*-test.

Data are presented as number of observations (*n*) and means ± standard deviation.

**Table 4:** Correlation analysis of parameters of body composition with cardiovascular and metabolic characteristics divided by sex in HL-IGR-patients.

	Body weight		BMI		Waist circumference	
	rho	*p* value	rho	*p* value	rho	*p* value
*HL-IGR-men*						
LDL-cholesterol	−0.10	NS	−0.13	NS	−0.10	NS
HDL-cholesterol	−0.14	NS	−0.19	NS	−0.04	NS
Total cholesterol	−0.00	NS	−0.05	NS	−0.03	NS
Triglycerides	0.16	NS	0.23	NS	0.06	NS
HbA1c	−0.00	NS	−0.13	NS	0.17	NS
Fasting plasma glucose	0.08	NS	0.01	NS	0.18	NS

*HL-IGR-women*						
LDL-cholesterol	−0.11	NS	−0.14	NS	−0.01	NS
HDL-cholesterol	−0.24	NS	−0.21	NS	−0.07	NS
Total cholesterol	−0.09	NS	−0.07	NS	0.06	NS
Triglycerides	0.35	NS	0.36	NS	**0.49**	**0.04**
HbA1c	−0.02	NS	0.25	NS	0.11	NS
Fasting plasma glucose	0.10	NS	0.16	NS	0.33	NS
